# *TAS2R38* and Its Influence on Smoking Behavior and Glucose Homeostasis in the German Sorbs

**DOI:** 10.1371/journal.pone.0080512

**Published:** 2013-12-02

**Authors:** Maria Keller, Xuanshi Liu, Tobias Wohland, Kerstin Rohde, Marie-Therese Gast, Michael Stumvoll, Peter Kovacs, Anke Tönjes, Yvonne Böttcher

**Affiliations:** 1 IFB AdiposityDiseases, University of Leipzig, Leipzig, Germany; 2 Bioinformatics Group, Department of Computer Science, University of Leipzig, Leipzig, Germany; 3 Department of Medicine, University of Leipzig, Leipzig, Germany; German Institute of Human Nutrition Potsdam-Rehbruecke, Germany

## Abstract

**Background:**

Genetic variants within the bitter taste receptor gene *TAS2R38* are associated with sensitivity to bitter taste and are related to eating behavior in the Amish population. Sensitivity to bitter taste is further related to anthropometric traits in an genetically isolated Italian population. We tested whether the *TAS2R38* variants (rs713598; rs1726866 and rs10246939) may be related to eating behavior, anthropometric parameters, metabolic traits and consumer goods intake in the German Sorbs.

**Materials and Methods:**

The three SNPs were genotyped in a total cohort of 1007 individuals (male/female: 405/602). The German version of the three-factor eating questionnaire was completed by 548 individuals. Genetic association analyses for smoking behavior, alcohol and coffee intake, eating behavior factors (restraint, disinhibition and hunger) and other metabolic traits were analyzed. Further, by combining the three SNPs we applied comparative haplotype analyses categorizing PAV (proline-alanine-valine) carriers (tasters) vs. homozygous AVI (alanin-valine-isoleucine) carriers (non-tasters).

**Results:**

Significant associations of genetic variants within *TAS2R38* were identified with percentage of body fat, which were driven by associations in women. In men, we observed significant associations with 30 min plasma glucose, and area under the curve for plasma glucose (0–120 min) (all adjusted *P*≤0.05). Further, we found that carriers of at least one PAV allele show significantly lower cigarette smoking per day (*P = *0.002) as well as, albeit non-significant, lower alcohol intake. We did not confirm previously reported associations between genetic variants of *TAS2R38* and eating behavior.

**Conclusion:**

Our data suggest that genetic variation in *TAS2R38* is related to individual body composition measures and may further influence consumer goods intake in the Sorbs possibly via individual sensitivity to bitter taste.

## Introduction

The human gustatory sense is mainly mediated by different taste receptor cells (TRCs) clustered in taste buds predominantly located on the tongue [1]. Further, bitter taste receptors are also expressed in other parts of the gastrointestinal tract [2]. Water-soluble chemicals interact with the apical ends of TRCs resulting in activation of downstream signaling pathways and transmission of signals to the central nervous system [1], [2]. The ability to taste bitterness significantly influences human eating behavior via the development of individual food preferences [2]. In humans, more than 30 different bitter taste receptors have been identified [3]. These G protein-coupled receptors of the TAS2R family are expressed in TRCs on the tongue and in cells of the gastrointestinal mucosa and are involved in energy and glucose metabolism [2], [3]. Sensitivity to bitterness varies between individuals and is associated with genetic variants within genes encoding bitter taste receptors. One of these genes is the well characterized *TAS2R38* [4]. Three single-nucleotide polymorphisms (SNPs) (rs713598; rs1726866 and rs10246939) within the *TAS2R38* gene, resulting in three amino acid substitutions (A49P; A262V and V296I), are primarily responsible for individual variability in bitter taste perception in response to phenylthiocarbamide (PTC) and 6-n-propyl-2-thiouracil (PROP) [2], [4]. Only two common haplotypes Ala-Val-Ile (AVI) and Pro-Ala-Val (PAV) are known outside sub-Saharan Africa [5], [6]. Heterozygous and homozygous PAV haplotype carriers are more sensitive to PTC and PROP [6], [7] compared to AVI/AVI-carriers. This allows one to classify individuals as tasters or non-tasters based on their genotype [6], [8]. It has been shown that SNPs within *TAS2R38* (rs1726866) are nominally associated with the eating behavior phenotype disinhibition in Amish women [8]. Furthermore, in a genetically isolated Italian population it has been shown that among women with low dietary restraint, those classified as non-tasters tend to have higher body mass index (BMI) and waist circumference (WC) than tasters [4].

In addition to the roles outlined above, the family of TAS2R receptors may modulate digestive behavior and glucose homeostasis via signaling pathways e.g. of glucagon like peptide-1 (GLP-1) which is a modulator of insulin biosynthesis and secretion [9], [10]. Dotson and colleagues have shown that genetic variation within several bitter taste receptors significantly influences glucose homeostasis [4], [8].

Lastly, the sensitivity to bitter taste may influence alcohol intake [11] and smoking behavior [12]. As demonstrated by Duffy et al. 2004 [11] homozygous PAV carriers show lower alcohol intake compared to individuals homozygous for AVI. In line with this, Mangold et al. 2008 [13] observed a higher smoking quantity among non-tasters compared to tasters. Further, Cannon et al. 2005 [14] reported differential haplotypic associations and smoking behavior.

Here, we describe the analysis of three *TAS2R38* variants and the corresponding haplotypes in a self-contained German population, the Sorbs, in regard to consumer goods consumption as well as eating behavior phenotypes. To shed further light on a potential role of *TAS2R38* variants in glucose metabolism, we tested for genetic association with a wide range of metabolic phenotypes.

## Methods

### Subjects

The Sorbs cohort is a self-contained population from Eastern Germany which was extensively phenotyped for a wide range of anthropometric and metabolic phenotypes [15]. Phenotyping included standardized questionnaires for individual medical history, family histories and eating behavior factors (German version of three factor eating questionnaire, FEV) [16]. Weight, height, waist-to-hip-ratio (WHR), body impedance analysis and a 75 g oral glucose tolerance test (OGTT) after an overnight fast were also included. The main characteristics of the study subjects are summarized in Table 1.

**Table 1:** Main characteristics of the study cohort.

	total	male	female	rs713598			rs1726866			rs10246939		
				CC	CG	GG	CC	CT	TT	GG	GA	AA
***N*** ** total**	1007	405	602	155	509	333	173	530	295	169	517	282
Age	48±16	48±16	48±15	48±15	47±16	49±17	48±16	47±17	50±16	48±16	47±16	50±16
BMI (kg/m^2^)	27.0±4.9	27.2±3.9	26.8±5.5	27.3±4.4	26.5±4.7	27.4±5.4	27.2±4.4	26.5±4.8	27.6±5.4	27.2±4.4	26.5±4.8	27.5±5.5
WHR	0.87±0.1	0.95±0.1	0.83±0.1	0.88±0.1	0.87±0.1	0.88±0.1	0.88±0.1	0.87±0.1	0.88±0.1	0.88±0.1	0.87±0.1	0.88±0.1
Body Fat (%)	21.3±9.2	18.0±6.9	23.5±9.9	22.0±8.4	20.5±8.5	21.9±10.3	21.9±8.3	20.7±8.9	22.1±10.2	21.9±8.4	20.6±8.9	22.0±10.3

Data are presented as mean ± SD. Reference allele according to HapMap database, standardized to the reverse strand: rs713598 G; rs1726866 C; rs10246939 A. BMI: body mass index; WHR: waist-to-hip ratio.

Individuals with type 2 diabetes (T2D) were excluded when analyzing eating behavior factors and phenotypes of glucose metabolism. A total of 1007 subjects (male/female: 405/602) with mean age of 48±16.2 years and mean BMI 27.0±4.9 kg/m^2^ were included. All subjects gave written informed consent and the study was approved by the ethics committee of the University of Leipzig.

### German Version of the Three-factor Eating Questionnaire

Of the total cohort 548 sorbs completed the German version of the three-factor eating questionnaire (FEV) [16]. The questionnaire measures the factors (dietary restraint, disinhibition and hunger) influencing individual eating behavior, as described for the Sorbs elsewhere [17].

### Genotyping

Genomic DNA was extracted using QIAmp DNA Blood Midi Kit (Qiagen Inc., Valencia, CA, USA) according to manufactureŕs instructions. Genotyping of rs1726866 was performed using TaqMan® SNP Genotyping Assay (Applied Biosystems by Life-Technologies Carlsbad, CA, USA). Rs713598 and rs10246939 were genotyped using the KASPar genotyping system (KBioscience allele specific PCR Genotyping System; KBioscience, Teddington, Middlesex, UK). Fluorescence was detected by an ABI 7500 Real-Time PCR system.

### Quality Control

All SNPs were in Hardy-Weinberg equilibrium (all *P*>0.05) To avoid genotyping errors, a random selection (∼5%) of the sample was re-genotyped; all genotypes matched the initially designated genotypes. Water was used as a no template control (NTC). Of the NTCs, 100% were determined as empty samples.

### Statistics

Non-normally distributed data were logarithmically transformed to approximate a normal distribution. Linear regression models adjusted for sex, age and lnBMI were employed to test for association of the three SNPs/haplotypes with eating behavior factors (restraint, disinhibition and hunger), anthropometric traits as well as glucose and insulin metabolism phenotypes. Moreover, sex stratified analyses were conducted. Linear regression models corrected for sex and age were applied to analyze consumer goods consumption (coffee, smoking and alcohol intake). Coffee consumption was defined as cups per day, smoking behavior as number of cigarettes per day and alcohol intake as number of glasses per week. Additive model of inheritance was tested. *P*-values <0.05 were regarded as providing nominal evidence for association. Given the directed “a priori hypotheses” of the study results were not corrected for multiple comparisons. Haplotypes were reconstructed for population genotype data using PHASE v2.1.1 [18] raised by rs713598 (A49P), rs1726866 (A262V) and rs10246939 (V296I). Three haplotypes were mainly detected: AVI [alanine-valine-isoleucine (55.9%)], PAV [proline-alanine-valine (40.9%)] and AAV (alanine-alanine-valine (2.9%)]. SPSS statistics version 20.0.1 (SPSS, Inc.; Chicago, IL) was used for all statistical analyses.

## Results

### Association with Anthropometric Measures and Glucose Metabolism

Using linear regression analysis we identified significant associations between the three *TAS2R38* genetic variants and percentage of body fat in non-diabetic women (all *P*≤0.05, Table 2). Minor allele carriers showed lower body fat content compared to homozygous major allele carriers (Table 2). No significant relationships to BMI and other anthropometric measures, such as WHR, were found. Furthermore, albeit non-significant, we observed slightly decreased BMI and body fat content in PAV haplotype carriers (PAV/PAV+PAV/AVI) compared to homozygous AVI haplotype carriers (Table 3).

**Table 2:** Association analysis of TAS2R38 SNPs with anthropometric measures and phenotypes of glucose and insulin homeostasis.

	*TAS2R38 Genotype*								
	rs713598			rs1726866			rs10246939		
Genotype (*N*)	CC (137)	CG (460)	GG (294)	CC (153)	CT (478)	TT (261)	GG (149)	GA (467)	AA (249)
* N* male	54	185	116	59	194	100	57	190	94
* N* female	83	275	178	94	284	161	92	277	155
BMI (kg/m^2^)									
male	26.9±3.3	26.5±3.5	27.1±3.6	26.9±3.3	26.5±3.6	27.2±3.4	26.8±3.4	26.4±3.4	27.2±3.8
female	26.7±4.7	25.9±5.2	26.7±5.7	26.6±4.6	25.9±5.2	26.8±5.7	26.7±4.7	25.9±5.3	26.7±5.7
p-value	0.873°; 0.939*			0.947°; 0.534*			0.723°; 0.627*		
Body Fat (%)									
male	17.4±6.0	16.8±5.8	17.2±5.9	17.4±6.0	16.9±5.9	17.8±6.1	17.3±6.1	16.8±5.8	17.9±6.5
female	23.8±8.3	22.4±9.2	23.2±10.7	23.5±8.2	22.4±9–4	23.3±10.8	23.7±8.3	22.4±9.4	23.0±10.7
p-value	**0.023°;** 0.909*			**0.013°;**0.680*			**0.012°;** 0.832*		
WHR									
male	0.94±0.08	0.93±0.08	0.94±0.08	0.94±0.08	0.93±0.08	0.94±0.08	0.94±0.08	0.93±0.08	0.95±0.09
female	0.83±0.07	0.81±0.08	0.82±0.07	0.83±0.07	0.81±0.07	0.83±0.07	0.83±0.07	0.81±0.07	0.82±0.07
p-value	0.938°; 0.368*			0.670°; 0.993*			0.852°; 0.963*		
Fasting Plasma Glucose (mmol/L)									
male	5.44±0.5	5.46±0.5	5.43±0.5	5.44±0.4	5.46±0.5	5.46±0.4	5.41±0.4	5.44±0.5	5.46±0.4
female	5.17±0.5	5.13±0.5	5.21±0.6	5.14±0.4	5.14±0.5	5.22±0.6	5.17±0.5	5.14±0.5	5.21±0.6
p-value	0.517°; 0.448*			0.478°; 0.668*			0.757°; 0.979*		
Plasma Glucose 30 min (mmol/L)									
male	9.13±1.8	9.05±1.6	8.71±1.4	9.08±1.7	9.06±1.6	8.72±1.4	9.02±1.8	9.00±1.6	8.75±1.4
female	7.99±1.5	8.00±1,6	8.28±1.7	7.9±1.5	8.03±1.6	8.30±1.7	7.97±1.5	8.02±1.6	8.26±1.6
p-value	0.168°; **0.045** *			0.123°; 0.062*			0.224°; 0.161*		
2 hr Plasma Glucose (mmol/L)									
male	5.39±2.0	5.23±1.8	5.00±1.7	5.41±2.1	5.11±1.8	5.08±1.8	5.40±2.1	5.08±1.8	5.08±1.8
female	5.78±1.7	5.44±1.7	5.71±1.6	5.72±1.7	5.44±1.7	5.75±1.6	5.78±1.6	5.40±1.7	5.77±1.6
p-value	0.836°; 0.052*			0.747°; 0.153*			0.759°; 0.979*		
Fasting Plasma Insulin (pmol/L)									
male	40.07±25.9	36.55±22.8	36.36±21.3	39.42±25.2	36.45±22.5	37.14±23.0	38.92±25.1	36.33±22.6	37.27±22.9
female	37.87±18.7	38.26±23.5	41.71±25.0	37.03±17.6	38.85±23.9	42.09±25.4	37.60±18.0	39.15±24.1	41.46±25.4
p-value	0.314°; 0.150*			0.226°; 0.109*			0.402°; 0.172*		
Plasma Insulin 30 min (pmol/L)									
male	273.8±154.0	283.9±168.1	294.3±208.5	275.6±153.4	289.4±167.4	291.2±220.1	267.5±150.8	289.6±167.9	295.8±222.7
female	284.4±141.5	300.7±179.5	298.8±197.1	289.8±151.1	300.2±182.0	299.1±196.0	291.3±149.8	303.5±185.0	291.6±194.3
p-value	0.898; 0.910*			0.972°; 0.403*			0.609°; 0.783*		
2 hrs Plasma Insulin (pmol/L)									
male	176.1±225.8	144.4±160.8	128.3±141.7	173.4±218.7	140.2±160.4	135.9±155.1	167.8±219.8	139.7±160.8	136.7±155.4
female	197.7±133.6	187.6±147.6	195.4±139.2	195.3±128.7	187.3±147.1	205.3±166.3	198.4±127.8	189.9±161.4	196.2±143.1
p-value	0.754°; 0.162*			0.839°; 0.263*			0.471°; 0.416*		
AUC Glucose 0–120 min (mmol/L)									
male	14.53±2.3	14.34±2.5	13.82±2.2	14.50±2.3	14.26±2.5	13.89±2.2	14.43±2.3	14.18±2.4	13.92±2.3
female	13.61±2.6	13.36±2.4	13.87±2.6	13.48±2.5	13.39±2.5	13.93±2.6	13.59±2.5	13.35±2.5	13.90±2.5
p-value	0.266°; **0.003** *			0.191°; **0.013** *			0.291°**; 0.032** *		
AUC Insulin 0–120 min (pmol/L)									
male	397.8±274.2	401.5±233.5	416.0±289.1	415.6±279.5	403.9±232.1	402.4±290.5	403.2±278.7	403.6±233.3	407.7±298.7
female	456.7±245.3	451.7±250.9	442.1±208.6	445.5±211.7	450.1±248.6	463.6±264.9	449.5±209.0	455.8±260.4	449.1±248.1
p-value	0.820°; 0.377*			0.845°; 0.226*			0.698°; 0.415*		

Data are presented as mean ± SD. Type 2 diabetics were excluded. *P*-values were calculated using additive model of inheritance using linear regression adjusted for age, sex and lnBMI (except for BMI); ° = female;

* = male; BMI: body mass index; WHR: waist-to-hip ratio; AUC: area under the curve. Reference allele according to HapMap database, standardized to the reverse strand: rs713598 G; rs1726866 C; rs10246939 A.

**Table 3:** *TAS2R38* haplotypes: anthropometric measures and phenotypes of glucose and insulin homeostasis.

	AVI/AVI	PAV/AVI+PAV/PAV	*P*-value
***N*** ** total (m/f)**	262 (111/151)	572 (218/354)	
BMI (kg/m^2^)	26.7±4.8	26.2±4.5	0.379
Body fat (%)	20.6±9.3	20.5±8.4	0.056
WHR	0.87±0.09	0.86±0.09	0.724
Fasting plasma glucose(mmol/L)	5.31±0.58	5.25±0.48	0.654
Plasma glucose 30 min(mmol/L)	8.59±1.58	8.33±1.67	0.112
2 hr plasma glucose(mmol/L)	5.56±1.75	5.32±1.72	0.066
AUC glucose(0–120 min)	14.10±2.46	13.65±2.48	**0.036**
Fasting plasma insulin(pmol/L)	38.63±22.63	38.00±23.54	0.994
Plasma insulin 30 min(pmol/L)	288.54±195.92	291.65±170.34	0.250
2 hr plasma insulin(pmol/L)	176.16±160.83	169.73±150.12	0.484
AUC insulin(0–120 min)	430.27±255.47	428.32±241.24	0.672

Data are presented as mean ± S.D. Type 2 diabetics were excluded. *P*-values were calculated using linear regression adjusted for age, sex and lnBMI (except for BMI). AVI = alanine-valine-isoleucine; PAV = proline-alanine-valine, BMI: body mass index; WHR: waist-to-hip ratio; AUC: area under the curve.

Next, we tested whether *TAS2R38* genetic variants influence phenotypes of glucose and insulin metabolism. In non-diabetic men, we found a relationship between genetic variants and 30 min plasma glucose levels as well as area under the curve (AUC) of plasma glucose (0–120 min). We observed increased AUC and glucose levels among minor allele carriers, applying additive model of inheritance (adjusted for sex, age and lnBMI, Table 2). Similar results were obtained when adjusting for age, sex and percentage of body fat. Moreover, we observed decreased 30 min plasma glucose levels and AUC values in PAV allele carriers (Table 3). No relationship was detected between *TAS2R38* haplotypes and markers of insulin metabolism.

### TAS2R38 May Influence Smoking Behaviour, Alcohol and Coffee Consumption

**Figure 1:**
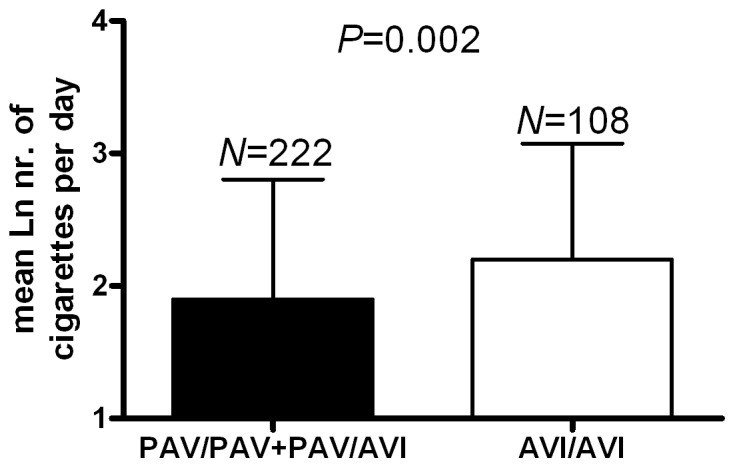
*TAS2R38* haplotypes and smoking. Data are presented as mean ± S.D. *P*-value was calculated using linear regression adjusted for age and sex. AVI = alanine-valine-isoleucine; PAV = proline-alanine-valine.

We tested for associations between SNPs within *TAS2R38* and smoking behaviour. No significant relationship was observed. However, carriers of at least one PAV haplotype showed significantly decreased cigarette smoking per day compared to homozygous AVI-carriers (Figure 1, Table 4).

**Table 4:** *TAS2R38* haplotypes and consumer goods.

	AVI/AVI	PAV/AVI+PAV/PAV	*P*-value
***N***	108	222	
smoking	12.5±10.1	9.3±6.9	0.002
*N*	306	665	
alcohol	2.87±1.28	2.83±1.18	0.903
*N*	250	558	
coffee	1.82±0.71	1.82±0.78	0.994

Data are presented as mean.± S.D. *P*-values were calculated using linear regression adjusted for age and sex. AVI = alanine-valine-isoleucine; PAV = proline-alanine-valine.

We did not find effects of single genetic variants on alcohol consumption, however, categorizing individuals carrying at least one PAV haplotype vs. AVI/AVI homozygous carriers, we observed lower alcohol intake per week in the PAV group (Table 4). No associations were found for SNPs/haplotypes and coffee consumption.

### TAS2R38 and Eating Behaviour

Linear regression analyses for association of SNPs and haplotypes with the three different eating behaviour factors (restraint, disinhibition and hunger) did not show significant associations (Table S1). However, we observed a non-significant trend for increased restraint scores in PAV allele carriers as well as decreased disinhibition values (Table S2).

## Discussion

The main findings of the present study are: (i) a significant relationship of *TAS2R38* genetic variation was found with percentage of body fat in women and further, an association with phenotypes related to glucose homeostasis in men. Moreover, we observed (ii) that PAV haplotype carriers show significantly lower tobacco intake per day compared to homozygous AVI haplotype carriers. (iii) We did not confirm the previously reported significant relationship of genetic variation in *TAS2R38* with eating-related disinhibition [8].

Our findings in the Sorbs cohort corroborate the previously suggested relationship with body composition measures observed in the isolated residents of Carlantino in southern Italy [4]. Our results are also in line with glucose homeostasis associations demonstrated in the Amish Family Diabetes Study [19]. Besides the relationship between the *TAS2R38* variants and body fat content we also found that the three SNPs are related to glucose and insulin metabolism phenotypes as similarly reported for *TAS2R7* and *TAS2R9* [19]. While we assume these effects are mediated by the bitter taste receptor TAS2R38 on the tongue we cannot rule out the possibility that the receptors expression in other parts of the gastrointestinal tract plays a role and may, in part, mediate the observed relationships of genetic variants and quantitative traits [9], [20].

Supporting the hypothesis that glucose metabolism may be influenced by bitter taste sensitivity, PAV allele carriers showed consistently lower 30 min plasma glucose levels and AUC values compared to homozygous AVI carriers. While non-significant, we observed slightly decreased BMI and percentage of body fat in PAV haplotype carriers. Given that the PAV haplotype has been dubbed ‘PROP tasters’ [5], [6], [11] PAV allele carriers might be more sensitive to PROP tasting than non-carriers. Unfortunately, since PROP tasting sensitivity has not been tested in the Sorbs, we could not specifically test this hypothesis in our cohort. Nevertheless, individuals more sensitive to PROP or PTC (PAV allele carriers) may potentially show more selective eating behavior strategies. We found that individuals more sensitive to bitter taste show, albeit non-significant, higher restraint scores in eating behavior suggesting higher cognitive control. Thus, our data suggest the ability to taste bitterness might potentially influence glucose homeostasis via a more cognitive-based route (i.e. through eating behavior).

As previously reported by others, the ability to taste bitter compounds (PROP or PTC) predicted by *TAS2R38* haplotype (PAV) may also influence smoking and alcohol intake [11], [12], [13], [14]. We show that individuals carrying homozygous or heterozygous PAV haplotypes smoke significantly less cigarettes per day than homozygous AVI carriers, in line with the suggestion that these individuals avoid both bitterness and tobacco. Furthermore, our results are consistent with Duffy and colleagues [11], identifying decreased alcohol consumption in individuals carrying at least one PAV haplotype.

In regard to the eating behavior factors restraint, disinhibition and hunger we also confirmed the findings of Tepper et al. [4] which showed lower dietary restraint in non-tasting females. We find the same tendency for lower mean restraint values in the AVI/AVI group, which may be cautiously interpreted as individuals classified as non-tasters. However, we did not find any significant association between the *TAS2R38* SNPs or haplotypes and eating behavior factors. Further, we could not confirm the previously reported relationship between rs1726866 and reduced disinhibition scores in women from an Amish population [8]. However, we cannot entirely rule out that differences between the TFEQ [21] and FEV [16] regarding the interpretation of the disinhibition scale may have an impact on the relationship between genetic variation and eating behavior factors. When using the TFEQ there are difficulties in interpreting the factor disinhibition since the theoretical basis of disinhibition is not entirely clarified. According to Westenhöfer [22] disinhibited eating behavior requires former cognitively controlled eating behavior. Therefore, in the German version (FEV) the disinhibition scale is interpreted more as the interference of eating behaviour through emotional and situational circumstances eliminating this shortcoming of the TFEQ questionnaire. However, the same three dimensions of eating behavior factors are measured in both questionnaires.

Additionally, the current study also has several other limitations. The sample size of our cohort, especially the number of individuals with eating behavior data, is limited. Unfortunately, data were not available regarding phenotypic sensitivity to PROP or other bitter compounds, meaning a direct link between bitter tasting and consumer goods consumption cannot be made. Nonetheless, the data in regard to smoking and alcohol intake are in line with data previously reported [11], [12], [13].

In conclusion, our data suggest that, in Sorbs, genetic variation within *TAS2R38* may be related to anthropometric measures and glucose homeostasis. Moreover, the ability to taste bitterness may influence the amount of alcohol and tobacco intake.

## Supporting Information

Table S1 ***TAS2R38*** **genetic variants and eating behavior factors.** Data are presented as means ± SD. Type 2 diabetics were excluded. *P*-values were calculated using linear regression model adjusted for age, sex and lnBMI.
(DOC)

Table S2 ***TAS2R38*** **haplotypes and eating behavior factors.** Data are presented as means ± SD. Type 2 diabetics were excluded. *P*-values were calculated using linear regression model adjusted for age, sex and lnBMI. AVI = alanine-valine-isoleucine; PAV = proline-alanine-valine.
(DOC)
