# Supplementary material for: TAS2R38 and Its Influence on Smoking Behavior and Glucose Homeostasis in the German Sorbs
Source: PLoS One. 2013 Dec 2;8(12):e80512. doi: 10.1371/journal.pone.0080512 (PMC3846558; doi:10.1371/journal.pone.0080512)
Supplement: Table S1 — TAS2R38 genetic variants and eating behavior factors. Data are presented as means ± SD. Type 2 diabetics were excluded. P-values were calculated using linear regression model adjusted for age, sex and lnBMI. (DOC) [file pone.0080512.s001.doc]

**Table S1. *TAS2R38*** genetic variants and eating behavior factors.

|  | **Genotype** | **Gender** | | **Eating Behavior Factor** | | |
| --- | --- | --- | --- | --- | --- | --- |
|  | **(*N*)** | **male** | **female** | **Restraint** | **Disinhibition** | **Hunger** |
| **rs713598** | CC (90) | 36 | 54 | 7.8 ± 4.9 | 4.5 ± 3.3 | 4.0 ± 2.8 |
|  | CG (288) | 104 | 184 | 7.8 ± 4.7 | 4.2 ± 2.8 | 3.8 ± 2.8 |
|  | GG (166) | 61 | 105 | 8.2 ± 5.3 | 4.5 ± 3.2 | 4.0 ± 2.8 |
| p-value |  |  |  | 0.552 | 0.384 | 0.427 |
| **rs1726866** | CC (101) | 38 | 63 | 8.1 ± 4.9 | 4.6 ± 3.3 | 4.0 ± 2.7 |
|  | CT (289) | 105 | 184 | 7.7 ± 4.7 | 4.3 ± 2.8 | 3.9 ± 2.8 |
|  | TT (151) | 54 | 97 | 8.2 ± 5.1 | 4.4 ± 3.2 | 4.0 ± 2.9 |
| p-value |  |  |  | 0.994 | 0.899 | 0.470 |
| **rs10246939** | GG (96) | 36 | 60 | 8.2 ± 5.0 | 4.5 ± 3.2 | 3.9 ± 2.7 |
|  | GA (284) | 106 | 178 | 7.7 ± 4.7 | 4.3 ± 2.9 | 3.9 ± 2.8 |
|  | AA (143) | 49 | 94 | 8.1 ± 5.2 | 4.4 ± 3.2 | 4.0 ± 2.9 |
| p-value |  |  |  | 0.820 | 0.657 | 0.404 |

Data are presented as means ± SD. Type 2 diabetics were excluded. *P*-values were calculated using linear regression model adjusted for age, sex and lnBMI.
