# Supplementary material for: TAS2R38 and Its Influence on Smoking Behavior and Glucose Homeostasis in the German Sorbs
Source: PLoS One. 2013 Dec 2;8(12):e80512. doi: 10.1371/journal.pone.0080512 (PMC3846558; doi:10.1371/journal.pone.0080512)
Supplement: Table S2 — TAS2R38 haplotypes and eating behavior factors. Data are presented as means ± SD. Type 2 diabetics were excluded. P-values were calculated using linear regression model adjusted for age, sex and lnBMI. AVI = alanine-valine-isoleucine; PAV = proline-alanine-valine. (DOC) [file pone.0080512.s002.doc]

**Table S2. *TAS2R38*** haplotypes and eating behavior factors.

|  | **AVI/AVI** | **PAV/AVI + PAV/PAV** | ***P*-value** |
| --- | --- | --- | --- |
| ***N*** | 155 | 359 |  |
| restraint | 7.77 ± 4.88 | 7.85 ± 4.79 | 0.980 |
| disinhibition | 4.52 ± 3.38 | 4.26 ± 2.85 | 0.237 |
| hunger | 4.03 ± 2.93 | 3.92 ± 2.83 | 0.531 |

Data are presented as means ± SD. Type 2 diabetics were excluded. *P*-values were calculated using linear regression model adjusted for age, sex and lnBMI. AVI = alanine-valine-isoleucine; PAV = proline-alanine-valine.
